# Decisions on the quality of piano performance: Evaluation of self and others

**DOI:** 10.3389/fpsyg.2022.954261

**Published:** 2022-11-17

**Authors:** Yuki Morijiri, Graham F. Welch

**Affiliations:** ^1^Graduate School of Teacher Education, Tokyo Gakugei University, Tokyo, Japan; ^2^UCL Institute of Education, University College London, London, United Kingdom

**Keywords:** performance criteria, pianists, self-evaluation, external-evaluation, piano performance

## Abstract

In common with other professional musicians, self-evaluation of practise and performance is an integral part of a pianist’s professional life. They will also have opportunities to listen to and evaluate the performances of others based on their own criteria. These self-constructed perspectives towards to a piano performance will have an influence on both self-evaluation and external evaluation, but whether differently or similarly is not known. Consequently, this research study aimed to explore how judgements on the perceived quality of a performance are undertaken by professional standard pianists and what criteria are applied, both with regards their own performances as well as the performance of others. Participants were six professional pianists (3 men, 3 women) who were based in the United Kingdom (Mean age = 31.5 years old. SD = 5.1). They were asked to play individually six trials of a piece of R. Schumann’s “Träumerei” Op. 15 No. 7 in a hired hall for recordings. Then, within 2 months, each participant was asked to come to a self-evaluation session to listen to and evaluate their own six recordings, using a Triadic method as a Repertory Grid. For the external evaluation focused session, the participants were asked to return again to evaluate a further six recordings made up of ‘best’ recordings as selected by each participant from their own individual self-evaluations. Analyses of the resultant data suggest that there was no significant difference between the participants in their overall ratings in the external phase, but that self-evaluation showed significant individual differences amongst several participants. The performance criteria in both self-evaluation and external evaluation predominately overlapped with each other in terms of musical factors, such as tone quality, phrasing, and pedalling. The ranking of the performances was highly correlated with perceptions of overall flow, tone quality and pedalling. It appears that pianists apply similar criteria to decide performance quality when evaluating their own performances as well as others.

## Introduction

Musical performances can vary greatly between individuals, and performers are reported to create an individual mental construction of their own performances (e.g., Sloboda, 2000; Repp and Knoblich, 2004). Performers have personal rules about performances and their own uniqueness of interpretation. Nevertheless, although different artists might express a piece of music differently from one another, the characteristics and distinguishing features are reported to be relatively usually maintained and stable in personal performances at the level of the individual (Jung, 2003; Lehmann et al., 2007). Performances by the same performer also tend to have an identity and oneness in terms of memory, action and performance parameters (Chaffin and Imereh, 2002; Gingras et al., 2011, 2013; Van Vugt et al., 2013). From the perspectives of listeners, performance characteristics are identifiable (Palmer et al., 2001), even by non-musicians (Koren and Gingras, 2014) and performer themselves (Keller et al., 2007). Therefore, it could be argued that individual differences, personal tendencies and personal rules become established through practise and experiences on the basis of their own sense of music and standards of music performance within the conventions of an established performance culture.

Earlier, Seashore (1938) reported that, in spite of each performer’s expression being different, the expressive parameters and variations are likely to be reproduced and maintained within the same performer. Moreover, Repp and Knoblich (2004) demonstrated that pianists were able to recognise their own recording amongst a set of several performances. In their research, they recorded 12 pianists playing 12 musical excerpts. Several months later, the researchers played these performances back and asked the pianists whether they thought that they were the person playing each excerpt. Participants gave their own performances significantly higher ratings than any other pianist’s performances. Furthermore, although they were presented with edited performances, which were different in tempo, overall dynamic level, and with dynamic nuances removed, the pianists’ accuracy ratings did not change significantly. This suggests that the remaining information was sufficient for self-recognition (Repp and Knoblich, 2004). The researchers concluded that “pianists seem to recognise their own performances because those performances create a stronger resonance in their action system than other performances do; this stronger resonance implies that there is a closer match between anticipated and perceived action effects” (Repp and Knoblich, 2004, p. 607). The results of this study suggested that although both playing and recognition of performance varied greatly between individuals, it seemed that there were individual rules on performance and inherent cognitive constructions in the performance evaluation. Furthermore, the implication of individuality in music performance is that performers have their own criteria in terms of performance and judge themselves and others on the basis of these criteria.

In terms of the nature of musical criteria used to evaluate a piano performance, these have been suggested in various ways (e.g., Abeles, 1973; Jones, 1986; Nichols, 1991; Palmer, 1996; Bergee and Cecconi-Roberts, 2002; Stanley et al., 2002; Zdzinski and Barnes, 2002; Juslin, 2003; Wapnick et al., 2005; Russell, 2010; Alessandri et al., 2016). For example, Duerksen (1972) suggested eight criteria for piano performance evaluation: rhythmic accuracy, pitch accuracy, tempo, accent, dynamics, tone quality, interpretation and overall quality. Russell (2010) suggested two broad categories, technical and musical, and five subordinate criteria for each: technical (tone, intonation, rhythmic accuracy, articulation and technique) and musical (tempo, dynamics, timbre, interpretation and musical expression). In other research, Thompson et al. (1998) explored how adjudicators rated the piano performance of different individuals by using Personal Construct Theory (Kelly, 1955), and in which these criteria were included: right-hand expression, phrasing, dynamics, rubato, form/structure, tone balance, pedalling, attention to rhythm and meter, articulation, technical competence, tempo, expression of several parts. In the same manner, other research studies have adopted other criteria (Duerksen, 1972; Saunders and Holahan, 1997). Several elements of classification used by one researcher appear to be redundant for another. In addition to the diversity of performance criteria, recent studies also underlined that there are difficulties and uncertainness in determining criteria of performance assessment (P. Johnson, 1997; McPherson and Thompson, 1998; McPherson and Schubert, 2004). Nevertheless, there has not been a consensus on performance criteria agreed by the research community, as each research study has used a different classification in order to evaluate performance. Moreover, the diversity of criteria and factors for performance evaluation have been discussed from the perspective of the comments by judges (Wrigley, 2005). It would be worthwhilst to explore the criteria that pianists themselves apply towards to their own performances to see if their viewpoints are different.

In the field of music performance, it has been argued that self-evaluation is one of the important processes in the development of performance skill from the perspective of self-regulation (Zimmerman, 2000; Zimmerman and Schunk, 2001; McPherson and Zimmerman, 2002). However, self-evaluations of music performances can often be inconsistent and biassed (Bergee, 1993; Kostka, 1997). In the process of self-evaluation, performers make judgements about whether their playing is good or needing improvement on their own terms and consider which elements that they might change and how (Brändström, 1996; Daniel, 2001). Even whilst playing, it is reported that performers will listen to and know their sense of the music, such as in terms of the stresses and phrasing, and be able to feed this knowledge back into the ongoing development of their own performances (Schmidt and Lee, 2010).

Self-assessment is a process of a formative assessment or evaluation of oneself or one’s action including performance, work, attitude and learning to an objective standard (Andrade and Du, 2007). Boud (1991) proposed the definition of self-assessment as “identifying standards and/or criteria to apply to their work and making judgements about the extent to which they have met these criteria and standards” (p.5). Andrade and Du (2007) provide a helpful definition of self-assessment that focuses on the formative learning that it can promote “during which people reflect on and evaluate the quality of their work and judge the degree to which they reflect explicitly stated goals or criteria, identify strengths and weaknesses in their work, and revise accordingly” (p.160). According to Boud (1995), all assessment, including self-assessment, comprises two key stages. The first is to develop knowledge, make decisions about the standards and criteria and apply them to a given work. The second is to assess critically the quality of the performance in relation to these criteria to see if it satisfies these standards or not. An engagement with setting the standards and their criteria are considered to underlie the process of learning (Boud, 1995).

Self-evaluation itself is known as a process of self-reflection and a potential enhancement of learning (McPherson and Zimmerman, 2002). Self-assessment can help to develop the skills effectively to monitor own performances and learning. Through a process of self-evaluation, a learner should be in a position to become more knowledgeable about how learning could be undertaken, what was learnt, how it would be judged and how it progressed, and also be able to utilise the outcomes into making a plan of how to improve further learning. Self-evaluation is considered an important part of ways to improve and enhance self-regulated learning (Boud, 1995). In other words, self-regulated learners are perceived as being more capable of monitoring themselves and of understanding the feedback that they receive and also engage in self-evaluation (McPherson and Zimmerman, 2002).

Whilst positive aspects of self-evaluation have been reported, some research studies have questioned the reliability and validity of self-evaluation (Gordon, 1991; Ross, 2006). For example, it has been demonstrated that self-evaluation does not always agree with instructors’ nor externals’ evaluations (Bergee and Cecconi-Roberts, 2002). Particularly, students’ evaluation and those by expert evaluators do not often match (Bergee, 1993; Kostka, 1997). Bergee (1997) demonstrated that the outcomes of students’ self-evaluation were less consistent, compared with faculty or peer evaluation, whilst also noting that there was no significant difference between the students’ level of self-evaluation performance and the type of instruments that they played. Several research studies have shown that there might be consistency, but also inconsistency with self-evaluation (Blatchford, 1997; Ross, 1998). For example, Kostka (1997) reported that self-assessment by piano students enrolled at the university compared to the assessment by their teachers showed relatively low agreement (students’ self-ratings were lower). Also, this research highlighted that self-assessment was influenced by students’ perceptions of what they “know,” namely self-perceptions of knowledge.

One of the reasons why self-evaluation can be difficult in terms of its reliability would be related to a feature of music evaluation. For self-evaluation, music performers are aware of their own performances during playing. Enhanced auditory feedback during performances can affect improvements in their performances (Repp, 1999; Finny and Palmer, 2003; Mathias et al., 2017). On the other hand, however, this kind of feedback might be problematic because the performers cannot listen to their own performance in the same ways as their audience (Daniel, 2001). In addition, it can be difficult for performers to evaluate their performance appropriately during the act of playing.

Moreover, complexities of performance evaluation itself include who makes the assessment. Some research studies have demonstrated that more experienced evaluators are likely to be more reliable in evaluation, whilst it might also be difficult to delineate between ‘more experienced’ or ‘more skilful’ and ‘less good’. The outcomes of research studies have been diverse. Whilst some research studies demonstrated that that more musically trained people are likely to have more reliable evaluation skills for music performances (Johnson, 1996; Shimosako and Ohgushi, 1996; Ekholm, 1997), several researches reported contrary findings that there is no substantial evidence to suggest that higher skilled musicians have more reliable assessment skills (Mills, 1987; Schleff, 1992; Bergee, 1993; Doerksen, 1999). However, it is agreed that the reliability and consistency on performance evaluation by trained musicians, such as professional musicians and faculty members, has been evidenced by research studies (Abeles, 1973; Thompson et al., 1998; Bergee, 2003; Ciorba and Smith, 2009). Wapnick et al. (1993) concluded that the relationship to the instruments which were the evaluator’s major study and which were related to the performance being assessed was not necessarily influential in reliable evaluation. This research study also suggested that “performers who excel in any one area of performance may excel in other areas as well” (p.283). It has been evident that non-musicians, who have not received formal musical training in higher education, or have very little prior experience in music, have a different perception and way of evaluating music performance (Geringer and Madsen, 1995/1996; Johnson, 1996). Therefore, it could be argued that higher musical and performance skills support the quality and reliability of musical assessment.

It has been suggested also that how people listen to and perceive music (e.g., Lerdahl and Jeckendoff, 1983; Madsen, 1990; Madsen et al., 1997) and how people evaluate performance as audiences (Johnson, 1996; Thompson et al., 1998; Hentschke and Del Ben, 1999) may be different from self-assessment in performance (e.g., Bergee, 1997; Daniel, 2001; Bergee and Cecconi-Roberts, 2002; Hewitt, 2011). It could be said that performers’ perspectives towards their own performances may be different from how others evaluate them. If performers have their own criteria and perspectives for self-evaluation, it would be worthwhile to investigate whether these same personal criteria are also used to judge the performances of others, or whether the self is a special case in terms of expectations. So far, the topic of whether each performer has two perspectives of performance evaluation, namely as a performer and as an audience when they listen to a performance, has been unexplored.

Therefore, this research study aimed to explore how the perceived quality of performance might be decided by professional standard pianists and what criteria were applied, both with regards their own performances as well as the performance of other peers. In particular, if each performer has a framework of criteria for performance evaluation, it is worthwhile to explore how each musical framework element contributes to the decision concerning the quality of piano performance. Also, how their own constructs could affect both self-evaluation and an evaluation of the performances by others.

## Methodology

### Personal construct theory and a triadic method

In order to identify personal constructs as an interpretation of person’s experience, a large range of research studies, including in clinical settings, education and the arts, have applied a Repertory Grid Technique which was suggested originally by Kelly (1955) (e.g., Beail, 1985; Saúl et al., 2012) in his personal construct theory (PCT). This was based on a concept that “a person’s processes are psychologically channelized by the way in which he anticipates events” (Kelly, 1955, p. 46).

In the field of music education and music psychology, the application of PCT has been researched in relation to how people recognise and listen to music (e.g., Hargreaves and Colman, 1981; Thompson et al., 1998). Gilbert (1990) valued PCT as a way of eliciting people’s insights as individuals and also of how the elicitation of constructs could have a prospective value for researching how people recognised and developed their own musical perceptions. Thompson et al. (1998) demonstrated constructs of piano performance criteria by six adjudicators with six recordings of a Chopin’s Etude. The researchers suggested that, by using a repertory grid technique, adjudicators could develop and refine their skills in evaluation by recognising insights and comparing their personal constructs. Consequently, the method of PCT is seen as not only a useful way to elicit personal constructs from an individual, but can also be beneficial in the development of the person who is involved in the process of using the technique in terms of understanding their own inner vision of a certain world.

Probably the most widely used PCT tool is the Repertory Grid Technique which is a method of eliciting constructs by asking participants to compare three elements and then stating how two are similar and different from the third. For example, in a musical context, two performances, A and B would be allocated a “slow” label and the other performance C could be “fast” on a construct called “tempo.” This procedure is called the “Triadic Method.” Answers are recorded in a matrix, which can then be analysed to produce a construct map. Regarding such a triadic method, Kelly (1955) originally suggested six ways that this could be applied: 1. The minimum context card form; 2. The full context form; 3. The sequential form; 4. The self-identification form; 5. The personal role form; and 6. Full context form with the personal role featured. The minimum context card form is the most widely used. This form provides three elements that are selected by the participants. The participants need to specify the important features in which two of the elements are similar and subsequently different from the third (Bannister and Mair, 1968; Fransella and Bannister, 1977). The pair of features given by the participant becomes a set of two construct poles, which is used in the next stage, namely completing a grid. In the current research study, the minimum context card form, which appeared to be the most common approach in the literature studies, was used as a basis for eliciting the personal constructs of participants.

Repertory Grid Technique is flexible as a methodology. However, it generally has five procedural stages: 1. Eliciting elements; 2. Eliciting constructs; 3. Completing the grid; 4. Analysis; and 5. Interpretation (Beail, 1985). “Elements can simply be provided by the investigator” or the researcher (Beail, 1985, p. 3). These can be places, people, and also can be generated by descriptions of a situation, unspecified acquaintances or giving roles (c.f. Fransella and Bannister, 1977; Beail, 1985). However, elements should “be representative of the area to be investigated” and “be within a particular range as constructs apply to only a limited number of people, events or things” (Beail, 1985, p. 4). In the current research, elements were recordings of piano performances by participants (c.f., Thompson et al., 1998).

Regarding the choice of constructs, there is another concern of whether or not these should be provided (Fransella and Bannister, 1977). From a practical perspective, to provide constructs can be vital, for example, when it is the purpose of the study to compare the relationship between verbal labels. From another point of view, supplied constructs can be given “a personal meaning by being related to those elicited” from the participant (Fransella and Bannister, 1977, p. 19). It cannot be always said that elicited constructs are more meaningful than provided constructs (Fransella and Bannister, 1977). A researcher needs to acquire a clear idea by understanding the participants’ recognition (Yorke, 1978). Kelly (1955) warned that verbal labels provided by the participants might not always reflect their innermost thoughts. Therefore, the researcher should know that the participants will attach their own meaning to the researcher’s label if a provided verbal label is used (Beail, 1985). Beail (1985) also added, “what is important is that a supplied verbal label be meaningful to the subject” (p.6). In this research, 13 musical criteria (overall flow, tone quality, interpretation of music, tempo, dynamics, rhythm, melodic accuracy, style, rubato, pedalling, technique, musical expression, phrasing) were listed as the suggested perspectives. These were elicited from previous music-focused research studies (e.g., Abeles, 1973; Jones, 1986; Nichols, 1991; Bergee and Cecconi-Roberts, 2002; Stanley et al., 2002; Zdzinski and Barnes, 2002; Juslin, 2003; Wapnick et al., 2005; Russell, 2010). However, the participants were also allowed to add their own ideas of criteria if they so wished. Providing some suggested construct options enabled the research to have a clear performance-focused context with appropriate musical criteria and acted as a framework for participants to understand their personal viewpoint in which they could also have the option of adding their own ideas of constructs.

**Figure 1:**
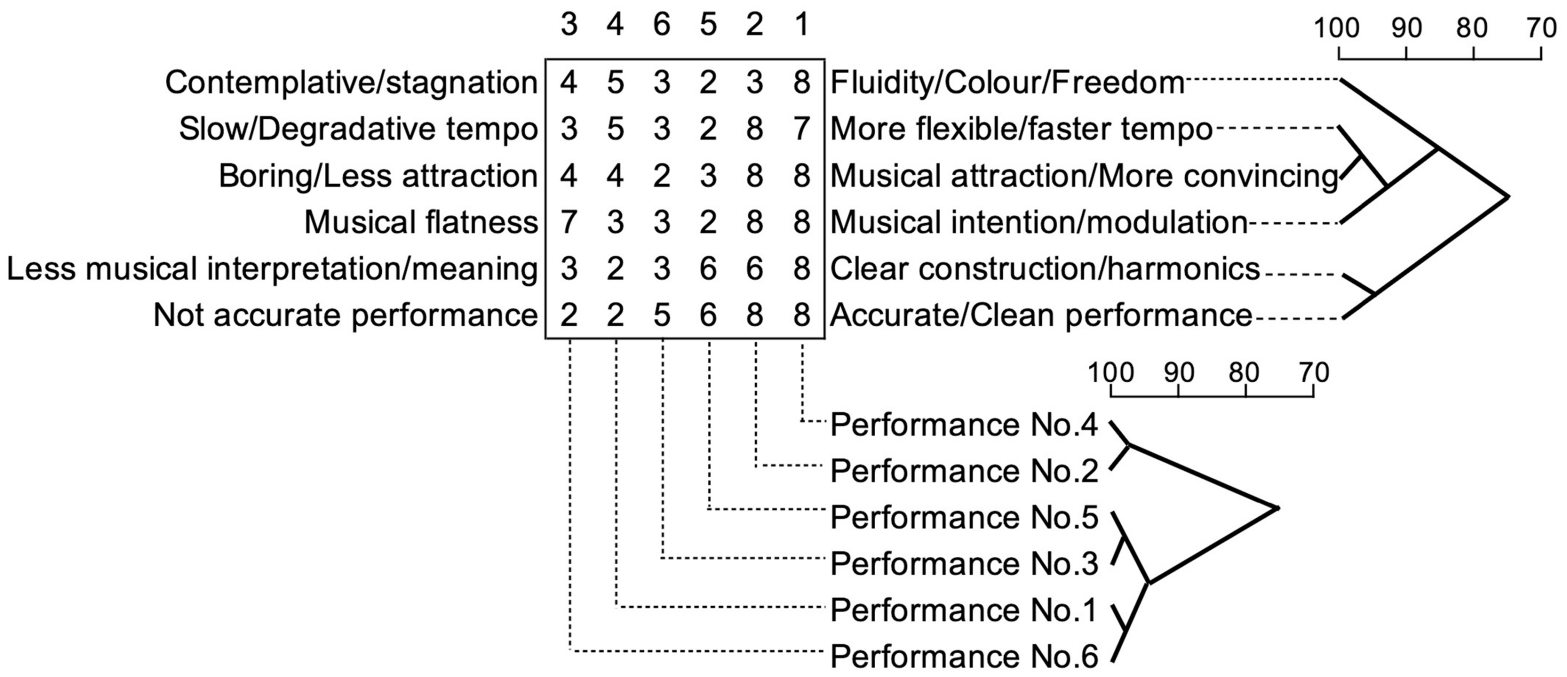
Sample grid from performer A.

In adopting this particular triadic method approach from repertory grid technique in the current research study, participants’ recordings of a selected piano piece were used as elements. Participants were tasked with choosing three recordings (as elements) and were asked to identify two similar features and a different feature. The participants then had to explain their choices – as an application of the triadic method – using a minimum context card form. Each participant was asked to name their construct, for example, with comments about “fast” versus “slow” on a construct called “tempo.” Figure 1 shows the sample grid with two construct poles for the six recordings. The participant could choose a construct from the provided list of 13 musical criteria, which were elicited from the previous research study, or to add another.

Analyses of the research literature suggests that, as well as there being different ways in eliciting constructs, there are also various ways of completing a grid. The current research study adopted a rating grid because “this method allows the person greater flexibility of response than does the rank grid” (Fransella and Bannister, 1977, p. 40). Based on the two construct poles given by a participant, each element was rated by how close it was to the description of the construct pole, for example, by using a seven point or nine-point scale. In this research study, a nine-point scale was adopted as it can be more precise as a measurable rating. In psychological research, having more scale points is thought to be better. However, there is a diminishing return after around 11 points (Nunnally, 1978).

### Participants

Participants were six professional pianists (3 men, 3 women, identified as Performers A–F) who were based in the United Kingdom (Mean age = 31.5 years, SD = 5.1 years). The mean duration of playing the piano was 27.7 years (SD = 4.9 years). In order to guarantee the professional expertise of the participants, the following conditions were required: to be a professional, active musician and concert pianist.

### Piece of music

In this research study, all participants were asked to play a common piece of music chosen by the researcher and to evaluate recordings of it for a research session. Robert Schumann’s “Träumerei” Op. 15, No. 7 was selected as the piece of music used. This piece is the one of the most famous and lyrical of Schumann’s piano pieces (Ostwald, 1985; Magrath, 1993; Gordon, 1996; Kapilow, 2011). This piece has been employed in several music psychological research studies regarding acoustic analysis and performance research (e.g., Repp, 1992; Friberg, 1995; Repp, 1995; Repp, 1996; Beran and Mazzola, 2000; Cambouropoulos and Widmer, 2000; Almansa and Delicado, 2009). It was also expected to encourage various individual differences in performances, as this feature has been reported in several previous research studies (e.g., Repp, 1992, 1995, 1996; Mazzola, 2011). From the perspective of its relative technical difficulty, this piece was selected for the syllabus of the 2007–2008 ABRSM Graded 7 Piano Exam (ABRSM, 2006). This suggests that Träumerei is not technically and musically easy; however, it not too difficult for professional pianists.

### Procedure

The research session had three phases: (i) Recording, (ii) Self-evaluation and (iii) External evaluation. The participants were asked to take part in all three sessions and to make sure that they understood all requirements and the schedule. They were informed of the research procedure by reading a specially designed research leaflet which also explained about confidentiality and related ethical concerns, as approved under the university’s ethical procedures for informed consent. The details of the three sessions were as follows.

#### The first session: Recording

The six participants were asked to practise a piece of Schumann’s “Träumerei,” Op. 15 No. 7 with repeats (c.f., Repp, 1992, 1994, 1995, 1996) before coming to the recording session. The participants were given a copy of the music score in advance, which was published originally by Breitkopf and Härtel (1839).

For the recording session, participants were asked to come to a hall at the University in London. The participants were asked to play six trials each of Schumann’s Träumerei with a tuned YAMAHA grand piano G5. Under the agreed ethical procedures, at the start of the session, each individual participant again received an explanation of the aims of the research and procedure and were asked to sign a research consent form. Before recording, the participants had 10 min to practise and to become familiar with the piano and to check the recording conditions for the sound recordist. With permission, six performances were recorded using an audio recording system (not video) with a professional recording engineer, and all recordings were made under the same conditions. Also, the participants were provided with a sheet to record their feelings about each trial (if they wished) and, at the end of the session, to report which performance that they thought was the ‘best’. The whole recording session for each person lasted ~45 min.

#### The second session: Self-evaluation

Within 2 months after making their original set of six recordings, each participant came to the Music Technology Suite at the University and listened to, evaluated, and made a comparison between their own six recordings. To do this, the participants again used a Triadic method with a minimum context card form (Fransella and Bannister, 1977; Wapnick et al., 1993). They received an explanation of the procedure and the purpose of the session before undertaking the evaluation.

Firstly, each participant was allowed to adjust the comfortable listening volume with headphones (Sennheiser HD650) using a VLC media player (version 2.0.6). The order of playback was from their original first performance to their sixth performance, and which was technically labelled as performance No. 1 to No. 6 in the session. There are some research studies which have showed that piano performances are highly likely to be receive higher scores in the latter order of playing (e.g., Duerksen, 1972; Flôres and Ginsburgh, 1996). The order of performance in both recording and self-evaluation was kept the same so that it could be determined if performance order could affect their decision on the quality of performance differently in both recording time and self-evaluation. Whilst listening to the six recordings, they were asked to write some notes on a comments sheet to be utilised later.

Secondly, after listening to all six versions, each participant was asked to choose three recordings (as elements) randomly, to compare them, identify two similar features and one different feature and then to explain their choices. Each participant was asked to name their features (label), write these down and to indicate each of three performances for each label. For example, two performances, No. 2 and No. 6, from the three could be allocated a “slow” label and the other performance, No. 4, could be labelled “fast” on a construct called “tempo.” The participant could choose a construct from the list of 13 musical criteria (overall flow, tone quality, interpretation of music, tempo, dynamics, rhythm, melodic accuracy, style, rubato, pedalling, technique, musical expression, phrasing), which were elicited from the previous research study, or add another of their choice. Each participant completed six sets of constructs. During this stage of evaluation, the participants were allowed to play back and listen to any recordings as many times as they wanted.

Thirdly, the participant rated each of their six recordings (the whole set) using a nine-point scale in terms of each of the six constructs that they had previously given. For example, if a participant named “slow” and “fast” in the construct for tempo, each of the six recordings was required to be rated as 1 = the fastest and 9 = the slowest. Because each participant arranged their six sets of constructs in this manner, all recordings were rated according to six sets of criteria. They were allowed to listen to the recordings again if they wanted. Finally, the participants ranked their six performances and were asked to choose the ‘best’ one (following Thompson et al., 1998). This whole session lasted ~1.5 h.

#### The third session: External evaluation

Within 2 months after this self-evaluation, the participants returned to the Music Technology Suite and evaluated a further six recordings. These were the six performers’ choices of their ‘best’ performance, including their own ‘best’ recording – although this was not disclosed until the end of the third session. Each performers’ personal information was not disclosed to participants.

Each participant was tasked with listening to the six recordings based on a randomised order (however, the adjudicator’s own recording was always placed as the 3rd), as indicated in Table 1. Listening took place under the same condition as in the second session in terms of the headphones (Sennheiser HD650) using VLC media player (version 2.0.6). The participant’s own recording was played as the third in the listening sequence for all participants. After listening to all recordings, the participants were asked to choose three recordings randomly, and to compare and apply the constructs as in the second session. They rated the six recordings based on the six sets of constructs. They also ranked the performances and chose the best performance amongst these six. These processes were the same as followed in the second session.

**Table 1:** The order of playback at the third session.

Adjudicator	Order of playback					
	1st	2nd	3rd	4th	5th	6th
Performer A	B	E	A	C	F	D
Performer B	C	F	B	D	A	E
Performer C	D	A	C	E	B	F
Performer D	E	B	D	F	C	A
Performer E	F	C	E	A	D	B
Performer F	A	D	F	B	E	C

A = the performance by Performer A. Performer’s own recording = shaded text.

At the end of the third session, the participant was informed that one of the recordings was their own performance and then was asked to identify if they knew which one this was, and to give a reason for their decision. Overall, this third and final session lasted ~1.5 h.

## Results

### Performance time

Table 2 shows the results of the length of each performance, which was measured from onset to offset of the performance sounds. The left column displays the trial number of each performance from the first to sixth performance. The total average length of performance was 2:35 min (range 2:03–3.33 min). The results of a repeated measures analysis of variance (ANOVA) showed a significant difference in terms of each performer’s mean time duration in performing the selected piece, *F*(5, 25) = 4.06, *p* = 0.008, η_p_^2^ = 0.45. This result indicated that strong individual differences were evident in performers’ playing (and, by implication, conception) of the music, even though all the performers played the same piece.

**Table 2:** The length of each performance.

Trials	Performer A	Performer B	Performer C	Performer D	Performer E	Performer F
1st	02:36	02:39	02:36	02:03	02:11	02:29
2nd	02:37	02:39	02:41	02:03	02:24	03:01
3rd	02:42	02:38	02:44	02:07	03:33	02:20
4th	02:44	02:42	02:44	02:02	02:04	***03:19***
5th	02:54	02:39	***02:40***	***02:09***	02:56	02:16
6th	***02:55***	***02:39***	02:38	02:03	***02:33***	03:12
Mean	02:44	02:39	02:40	02:04	02:36	02:46

Best perceived performance at the 1st recording session = italic and bold. Best ranked performance at the self-evaluation (2nd session) = shaded.

Performers E and F had some diversities regarding performance time for each of their trials. Performer E played with a range of 2:04–3:33 min and Performer F played with a range of 2:16–3:19 min. Both performers mentioned that they intentionally played each performance differently, based on what they wanted to express throughout each performance. In contrast, the other performers each tended to perform within a relatively more uniform time length.

Mauchly’s test indicated that the assumptions of sphericity had been taken violated, *X*(6) = 41.9, *p* < 0.001, therefore degrees of freedom were corrected using Greenhouse–Geisser estimates of sphericity (*ε* = 0.26). The results show that there was a significant effect in different time length of performance, *F*(1.31, 6.55) = 7.66, *p* = 0.039. These results suggested that there are individual differences amongst performers in terms of performance time. A pair-wise comparison between participants was undertaken to investigate which performers had mean time differences of performances. Significant differences are evidenced between performers D and A, B, C each (*p* < 0.01). Performer D had an average performance time of 2:04, which was the lowest average (the fastest tempo performance) amongst the six performers. Other pairs do not show significant differences. Performer D’s chosen recording was the shortest and Performer F’s recording was the longest, with a time difference of more than 1 min.

### Decision for the best performance as self-evaluation

At the recording-focused session (the first session), all participants were asked to think back over their examples and to decide which trial they thought to be the best. At the self-evaluation session (the second session) they listened to all of their own six recordings and ranked these from the best to least best recording. Table 3 shows performers’ choices of the best performance at the original recording session and also the subsequent self-evaluation session. At the recording, all performers reported that their best performance was in the latter half of their playing sequence, especially the fifth and sixth versions. In the self-evaluation session with audio playback, their choice of best performance might be the same or different. Overall, the matching of the choice of best performance between the initial session of recordings and the self-evaluation session was 50%. Participants were likely to choose the best performance from the latter half at both sessions.

**Table 3:** Choice of the best performance.

	At the 1st session (Recording)	At the 2nd session (Self-evaluation)*	Matching
Performer A	No. 6	No. 4	No
Performer B	No. 6	No. 6	Yes
Performer C	No. 5	No. 5	Yes
Performer D	No. 5	No. 3	No
Performer E	No. 6	No. 6	Yes
Performer F	No. 4	No. 6	No

*Recordings of the best performances at the 2nd session were actually used in 3rd session (external evaluation).

### The results of the self-evaluation

At the self-evaluation (second) session, each participant listened to their own six recordings, evaluated these by using a Triadic method for creating six sets of constructs, and rated each performance with a nine-point scale based on their six sets of constructs. Each recording (element) and construct was subjected to hierarchal cluster analyses with Ward’s method (1963).^1^ The analyses were undertaken by using SPSS.

**Figure 2:**
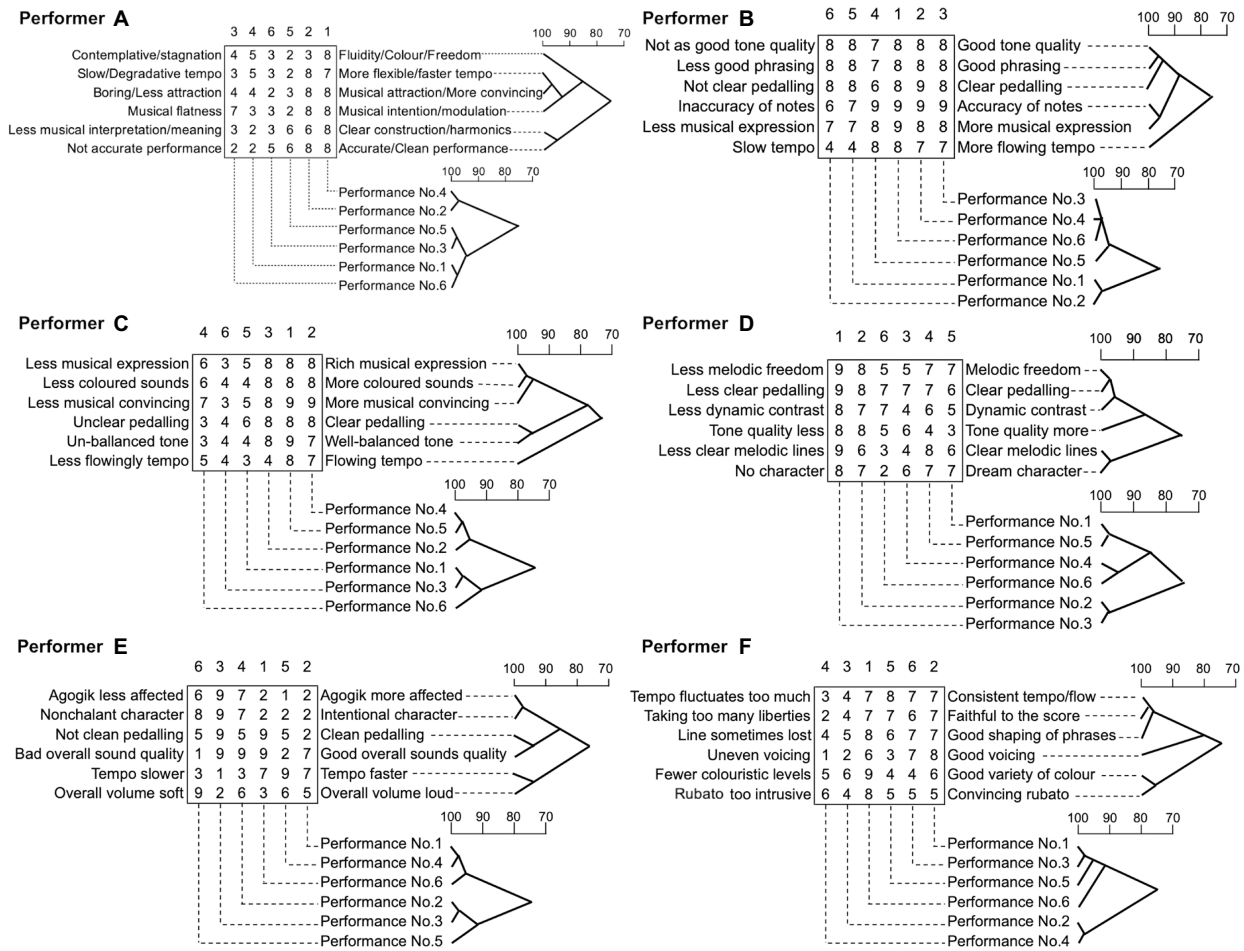
Clusters of performers’ self-evaluations of their own performances.

Figure 2A illustrates the result of the cluster analyses for both the constructs and the performances. This provides an illustration of the degree of association between constructs and between performances in a tree diagram. The top dendrograms shows the degree of association between constructs (poles). The 6 × 6 matrix of numbers presents the rating of each performance in each construct. Under the matrix, the dotted line indicates the number of the performance for each column. Above the matrix, the numbers show the ranking of performance, which displays as 1 (the highest rank) to 6 (the lowest rank).

As exampled in Figure 2A, each set of two performances which were of a closer ranking are strongly associated, No. 4 (rank 1) and No. 2 (rank 2) at 98%, No. 5 (rank 5) and No. 3 (rank 6) at 99% and then, No. 1 (rank 4) and No. 6 (rank 3) at 99%. The set of No. 5 and No. 3 is associated with the set of No. 1 and No. 6 at 94%. Performer A ranked Performance No. 4 as the best, which has the highest score in all constructs, apart from the sets of criteria ‘slow/degradative tempo’ and ‘more flexible/faster tempo’.

In particular, this ‘best’ performance attracted the highest score on the construct of ‘fluidity/colour/freedom’. Performance No. 3 was ranked as the lowest, which has the lowest rating in the construct pole of ‘boring/less attraction’ and ‘musical attraction/more convincing’. However, Performance No. 5 (rank 5) has more low-rated constructs than Performance No. 3. The constructs of ‘more flexible/faster tempo’ and ‘musical attraction/more convincing’ are most strongly associated at 99%. These two constructs are also associated with ‘musical intention/modulation’ at 93%. Similarly, the constructs of ‘less musical interpretation/meaning and clear construct/harmonics’ and ‘not accurate performance and accurate/clear performance’ are strongly associated at 98%. These two constructs show a low degree of association with other constructs at 75%.

These clusters were created for each participant in the same way as Performer A’s results. All illustrations are presented in Figures 2A–F. Figure 2B represents the result of a cluster analysis of the evaluation by Performer B. Performance No. 6 was chosen as the best as it achieved the highest total score. The top three performances (Performance No. 6, No. 4 and No. 3) were highly associated with each other at 99%. Performance No. 1 and No. 2 were also rated lower on the construct regarding tempo, which was associated with all other construct at 75%. The constructs of tone quality and phrasing were associated each other as the highest at 99%. These two constructs were also associated with the quality of pedalling at 98%. The degree of association with accuracy of note and musical expression was 97%.

Figure 2C displays the result of Performer C’s self-evaluation. On the constructions of criteria, balanced tone and pedalling were the most associated at 99%, as well as the combination of sound colour and musical expression, which were also strongly associated with being musically convincing at 98%. The construction of tempo was associated with all other constructs at 75%. Performance No. 5 ranked as the best overall and No. 4 ranked as the second best. These were strongly associated at 99%, as was Performance No. 3 which was ranked as the lowest with No. 1 ranked as the second lowest and associated at 99%. The best performance was the only one that got the highest rating amongst six performances on the construct of ‘Un/Well-balanced tone’ and ‘tempo’. Also, the top two performances, namely No. 5 and No. 4, achieved the highest rating on the criterion of ‘musically convincing’.

Figure 2D indicates the self-evaluation by Performer D. The top ranked Performance No. 3 achieved the highest score on all constructs and was associated with the second ranked Performance No. 2 at 98%. The lowest ranked Performance No. 6 got the lowest rating on the construction of ‘dream character’, which was associated with ‘clear melodic lines’ at 99%. The constructs of ‘melodic freedom’ and ‘clear pedalling’ were strongly associated at 99%, which were also related to ‘dynamic contrast’ at 96%. Performance No. 6 was the most associated with the third ranked Performance No. 4 at 94%. However, this third ranked performance recorded a lower score than the fourth ranked Performance No. 5, apart from the construct of tone quality.

Figure 2E shows the result of a cluster analysis of Performer E’s evaluation. Performance No. 6 was ranked the best and was strongly associated with Performance No. 4, ranked the second lowest at 99%. Surprisingly, the best performance did not have the highest ratings on all constructs. Compared with each rating on performances and the rank, it was not always likely to be consistent. The construct for rating, which is the most consistent in the ranking, seems to be sound quality. Tempo and the effect of ‘Agogik’ (which is a German word indicating the way of tempo changes) were highly associated with each other at 99%, as well as volume and pedalling being associated at 99%. ‘Character’ was associated with tempo and agogik at 83%. As performer E mentioned that he intentionally tried to perform differently on each trial, all recordings had various time ranges. It could be thought that tempo and agogik would be decided by what kind of character he would like to express. From the rating score, it could be said that more nonchalant character accompanies with the musical expression with less agogik (less tempo changes) in faster tempo.

Figure 2F displays the result of self-evaluation by Performer F. The best performance No. 6 marked the highest score, apart from the construction of ‘good voicing’ which gave the best score to the second ranked performance, No. 1. In particular, Performance No. 6 received the best score on the construct of ‘variety of colour’ which was consistent in the ranking. The constructions of ‘tempo’ and ‘faithful to the score’ were the most strongly associated at 99% and those of which were also associated with phrasing at 97%. The constructions of variety of colour and convincing rubato were associated with each other at 97%. The second-best performance No. 1 was more associated with Performance No. 3 ranked the lowest (99%) and No. 5 (95%) ranked the second lowest than the best performance (92%). The two lowest performances No. 3 and No. 5 did not overall record lower scores than the performances ranked the third and fourth. However, these two lower ranked performances were more deficient in ‘variety of colour’ which gave the highest score to the best performance No. 6. It seems that the construct of good voicing affects less on the overall ranking.

From all the results from the six pianists, there were three main conspicuous findings. The first finding was that the highest ranked recordings from each participant were likely to have obtained higher scores from certain criteria than others, such as in terms of musical expression, sound quality, phrasing and musical characteristics. The participants’ interviewed wording was varied; for example, to express tone quality, several criteria were revealed, such as more coloured sound, well-balanced tone and sound quality. In other words, the lowest ranked recordings had lower scores on these particular criteria even though the recordings obtained higher scores on other criteria.

The second finding was that time-related elements, namely tempo and rubato, were set as criteria in order to identify the characteristics of the performance. However, these did not always seem to affect a decision on the quality of performance. The focus piece of music, namely “Träumerei,” could be characterised as ‘slow and calm’ as it is the slowest piece of “Kinderszenen” (op.15) and demands the utmost in legato passagework. Nevertheless, several participants used words to describe the tempo as ‘flowing’, ‘consistent’ or ‘faster’.

The third finding was with regards to pedalling. The scores for pedalling were generally consistently related to the ranking of the recordings. However, several recordings had inconsistent scores in the rankings. It could be thought that pedalling would be an important component in deciding the quality of performance. However, other criteria such as musical expression, tone quality, phrasing and musical characteristics, were sometimes more dominant to determine the rankings.

Table 4 shows the results of overall self-evaluation ratings on their own six recordings by each participant. The rating range was from 1 to 9. The table indicates the mean rates and standard deviations. A one-way, between subjects, ANOVA was conducted to compare the difference of rating points on their own performances. There was a significant difference, *F*(5, 210) = 8.11, *p* < 0.001. *Post hoc* comparisons using the Turkey HSD test indicated that the mean score for the rating was significantly different between Performer B and all the others: B and A (*p* < 0.001), B and C (*p* = 0.03), B and D (*p* = 0.11) B and E (*p* < 0.001) and B and F (*p* < 0.001). Also, there was a significant difference between Performer A and Performer D (*p* = 0.09). It can be said that Performer B rated her own performances significantly higher than other performers did.

**Table 4:** Overall rating on self-evaluation.

Performer	A	B	C	D	E	F
Mean	4.78	7.64	6.11	6.36	5.28	5.50
SD	2.31	1.16	2.07	1.75	2.91	1.95

### The results of the external evaluation

At the end of the external evaluation session (the third session), all participants were informed that one of the recordings was their own performance and that this was the third in the sequence. Apart from Performer A, all other performers were able to identify their own recording amongst the six recordings used in the third session. The stated reasons why they could identify their own best recording were reported as following (as multiple answers): tone colour (2 reports), phrasings (2), dynamics (1), tempo (1) and no idea (1). Performer A declined to answer.

**Figure 3:**
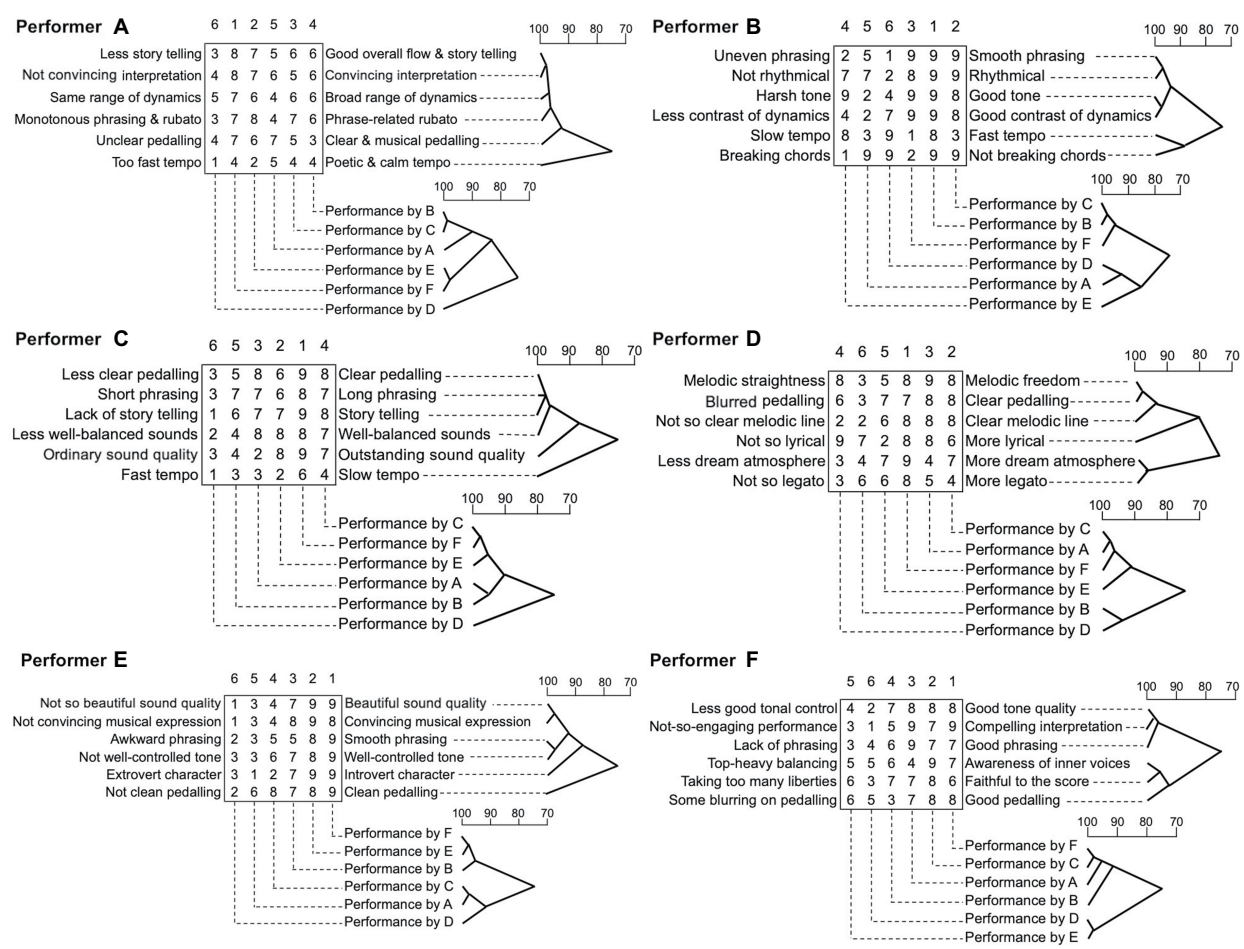
Clusters of performers’ evaluations of the performances of others.

Figure 3A illustrates the result of cluster analyses for both the constructs and the performances elicited by Performer A. This provides an illustration of the degree of association between constructs and between performances in a tree diagram. The top dendrograms shows the degree of association between constructs. The 6 × 6 matrix of numbers presents the rating of each performance in each construct. Under the matrix, the dotted line indicates the performer of the recording for each column. Above the matrix, the numbers show the ranking of the performance, which displays as 1 (the highest rank) to 6 (the lowest rank). Each performer’s best recordings used in the third session are displayed at the bottom of the matrix with dotted lines.

This Figure 3A also displays the result of a cluster analysis of the third session by Performer A. The recording performed by Performer F, which received the best score on the construct of “Convincing interpretation” and “Good overall flow & story telling”, was chosen as the best performance. The performance by Performer E, received the best score on the construct of ‘Phrase-related rubato’, and was ranked as the second-best performance. The constructions of ‘Convincing interpretation’ and “Good overall flow & story telling” were the most strongly associated at 99%. This cluster was also associated with ‘phrase-related rubato’ and ‘range of dynamics’ at 98%. The construction of ‘tempo’ was the least associated with others at 75%. Apart from ‘tempo’ and ‘pedalling’, other constructs were seen to be mostly consistent with the ranking. Performances played by Performer B (ranked fourth) and Performer C (ranked third) were the most strongly associated at 99% and were both associated with the performance by Performer A (ranked fifth) at 90%. The best-chosen performance by Performer F was the most strongly associated with the second-best version by Performer E at 99%. The performance by Performer D, which was the lowest ranked recording, was the least associated with the others at 75%. Performer A ranked her own recording as the fifth under the condition of disclosed information in which her recording was included. These clusters were created for each participant as same as Performer A’s results (Figures 3B–F).

The attribution of criteria seemed to be partly similar to the criteria in the self-evaluation. There were several noticeable findings as follows. For several participants, ‘interpretation’ seemed to be an important key feature in deciding the performance quality. As the participants listened to other pianists’ recordings, differences in interpretation were noticed and subject to comment. Yet, in the second phase self-evaluation, the ‘interpretation of music’ did not appear as a criterion, presumably because the listener (namely the performer) already knew their interpretation of the focused musical piece.

**Figure 4:**
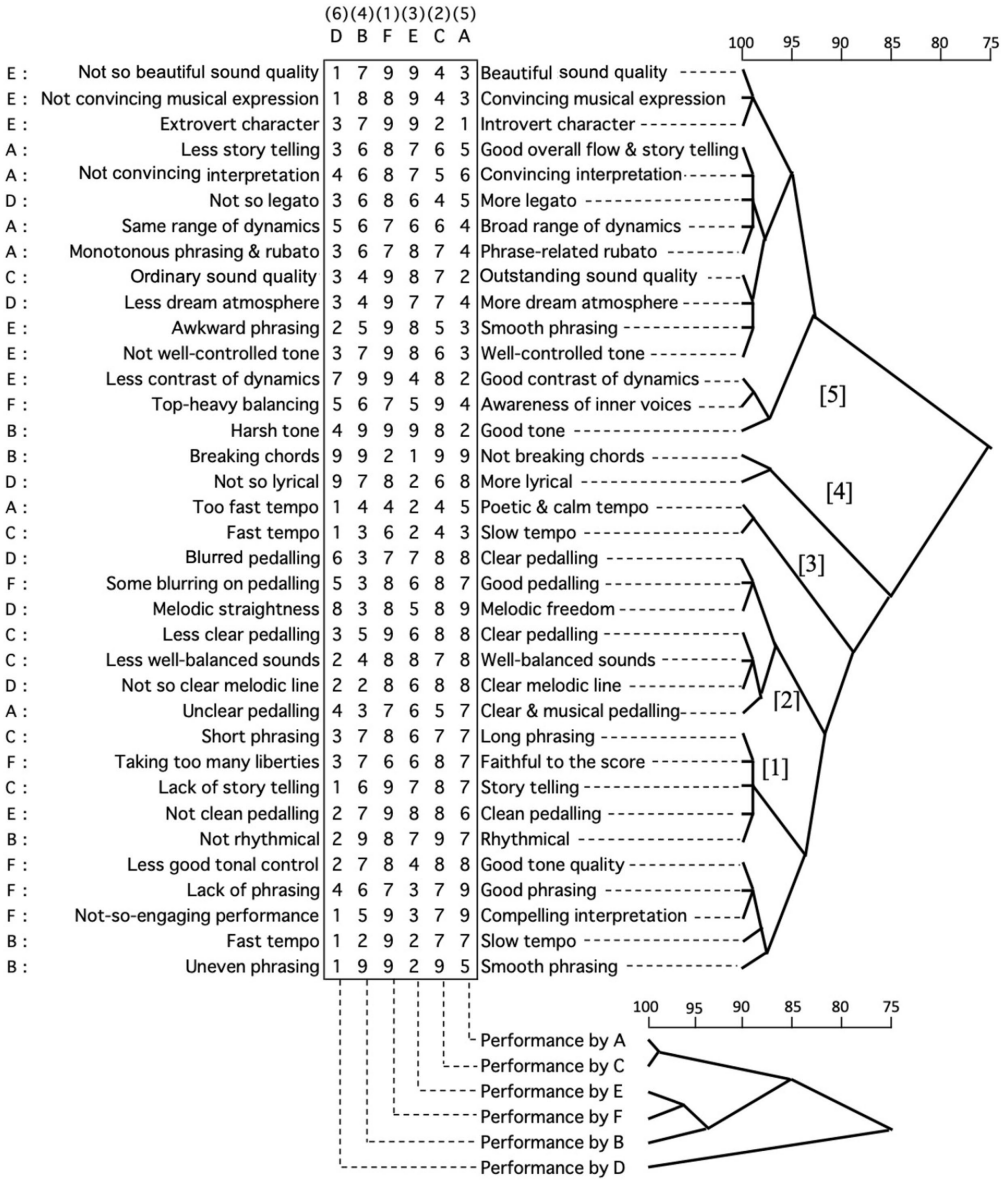
Cluster of all performance and constructs from external evaluation.

Overall, each participant’s data attracted different constructs in the evaluation and ranking of the performances. And each pianist had different perspectives and prioritisation in deciding which performance would be the best or highly ranked. However, the ratings did not always agree with ranking. This suggests that some criteria were being prioritised in the decision to choose a better/best performance. Although the participants ranking of the six performances were different, there were some underlying relationships evident amongst these six recordings in terms of their evaluations. Figure 4 illustrates the results of a cluster analysis using Ward’s method of all the constructs provided by all participants for the six recordings. The index of capital letters in the leftmost column is linked to which performer provided the set of constructs directly horizontal to it. For example, the first set of constructs, namely ‘not so beautiful sound quality’ and ‘beautiful sound quality’, were provided by Performer E. Numbers 1–9 inside the rectangular box display ratings for each performance as played by Performer A, B, C, D, E, and F in terms of the horizontal constructs. Above and below the ratings box, the capital letters indicate the performer of the recording for each column. The numbers with a round bracket above the capital letter indicates how the performer was ranked overall by all performers. For example, the leftmost column in the box indicates ratings for all constructs by all performers (evaluators) for the lowest ranked (the sixth rank) performance which was by Performer D.

The top dendrograms show the degree of associations amongst all the constructs provided by all performers (acting as the role of evaluators). The lower dendrograms indicate the degree of associations amongst all recordings used in the third session. The top dendrograms with all the constructs are divided into two large branches, which are associated with each other at 75%. The top branch consists of two sub-branches, which are connected with each other at 93%. These sub-branches contain lower sub-branches including several different musical perspectives as constructs; mainly, interpretation, tone quality, musical expression, and dynamics. The lower branch, which is associated with the top branch at 75%, shows a difference structure from the top one. In Figure 4 the branches of [1] and [2] contain the element of phrasing, and both of these are associated with another branch [3] at 91%, which includes the element of pedalling. These three branches are connected to the next branch [4] consisting of tempo at 89%. And then, they are associated with the branch [5] of ‘not breaking chords’ and ‘more lyrical’ at 85%. Compared with the two primitive branches divided at 75%, the top branch is likely to contain the constructs of more musical expression, tone quality and dynamics. On the other hand, the lower branch contains more focus on phrasing, tempo and pedalling. The construct related to interpretation appeared in both branches as well as musical atmosphere, for example ‘dream atmosphere’ and ‘story telling’.

The leftmost capital letters indicate the performers who provided the construct in a horizontal line, and also provides some ideas of the relationships amongst the performers giving the constructs. By focusing on the two large branches separated at 75%, the top branch contains all the constructs provided by Performer E. Also, this includes the four constructs provided by Performer A. Correspondingly; the lower branch comprises all the constructs assigned by Performer F and embraces the five constructs given by Performer C. In the top branch, the top three constructs were given by Performer E and were strongly associated with each other at 99%. Similarly, below these three constructs by Performer E, the four constructs assigned by Performer A were associated with each other at 99% in a branch. Also in the lower branch, the three constructs given by Performer F were highly associated with each other at 99%. Consequently, it could be said that several constructs provided by the same person were likely to be highly associated, although the constructs provided by Performers B and D seem to be separated more into both branches.

Regarding the recordings, the strongest association was between the performances by Performers A and C at 99%. The top ranked performance by Performer F was the most associated with the performance by Performer E at 96%, both of which were associated with the one by Performer B at 93%. The set of performances by Performers A and C is associated with the cluster constructed of performances by Performers E, F and B at 85%. The lowest ranked performance by Performer D was the least associated with all others at 75%.

The Supplementary Table 1 indicates the results of overall rating on external evaluation by each participant in terms of how they evaluated all six recordings acting as an adjudicator. The ratings range was from 1 to 9 and the table indicates the mean ratings and standard deviations. A one-way between subjects Analysis of Variance was conducted to compare the difference of the rating points in the external evaluation. There was no significant difference amongst the participants (*F*(5,210) = 0.779, *p* = n.s.). It could be said that their ratings behaviours were not affected by their individual differences and tendencies.

The Supplementary Table 2 indicates the results of the ranking of each performance at the external evaluation session. The rankings were converted as following: Ranking No. 1 = 6 points, No. 2 = 5 points, No. 3 = 4 points, No. 4 = 3 points, No. 5 = 2 points and No. 6 = 1 point. The left row shows each performer’s best performance used in the session and the column header shows evaluators, for example ‘A’ indicates Performer A. The matrix illustrates the converted points based on their rankings. The numbers in bold and underlined indicate the evaluator’s own performance. The mean points of each performance for the rankings are, ordered from the highest points to the lowest points: Performer F: 5.7, Performer C: 4.2, Performer E: 3.7, Performer B: 3.2, Performer A: 3.0, Performer D: 1.3. Overall, the performance by Performer F was ranked the best and that by Performer D was ranked as the lowest. The performance by Performer B was assigned the largest standard deviation. The agreement of ranking amongst evaluators was subjected to a Kendall Coefficient of Concordance analysis. The result suggested that the null hypothesis should be rejected at the 0.05 significance level (w = 0.584, *p* = 0.004). This implies that the evaluations of the selected recordings, including the evaluator’s own recording, could be concordant to some degree. Performer B and D evaluated their own performances relatively higher compared to the ratings by the other participants.

The participants provided six sets of criteria in order to evaluate the recordings in both the self-evaluation session and the subsequent external evaluation session. All constructions provided by the six pianists were categorised into the 13 original criteria (overall flow, tone quality, interpretation of music, tempo, dynamics, rhythm, melodic accuracy, style, rubato, pedalling, technique, musical expression, phrasing), which were elicited from previous research studies and provided to the participants as potential constructs. All constructs elicited by all the participants were categorised into these criteria and an analysis was undertaken using Spearman’s correlations to find the relationships amongst each element and ranking. The results indicated that there were significant correlations between: *Overall flow* and tone quality (*r* = +1.000), musical expression (*r* = +0.829), rubato (*r* = +0.899); *Tone quality* and musical expression (*r* = +0.829), rubato (*r* = +0.899); *Pedalling* and phrasing (*r* = +0.943), ranking (*r* = +0.829); and *Rubato* and ranking (*r* = +0.812).

Comparing the results from the self-evaluation and external evaluation, some criteria highly overlapped for each performer. The criteria used in these external evaluations included tone quality, phrasing, pedalling, tempi, and overall musical expression. An analysis using Kendall’s Coefficient of Concordance was undertaken in order to compare with both self and external evaluation criteria. The results revealed that they significantly overlap (*w* = 0.746, *p* < 0.001). It could be said that pianists in this research have similar constructs of criteria for the evaluation of piano performances, whether by themselves or by other pianists.

Regarding their decision concerning their best recording in both the recording session and self-evaluation session, the participants partly showed different decisions. At the recording session, Performer A, Performer B and Performer E decided that their sixth (final) recordings were thought to be the best, whilst Performers C and D chose their fifth performances. Performer F decided that the fourth one was their best. Data analyses showed that the participants chose their best recordings from their latter trials. In the self-evaluation session, Performers B, E and F chose their sixth recordings as the best. For other participants, Performer A chose the fourth recording and Performer C’s choice was the fifth one. Performer D decided that the third was the best. Matching of the decisions between the recording and the self-evaluation sessions was 50%. In each session, participants were likely to choose the best performance from the latter half.

## Discussion and conclusions

This research study aimed to explore how the perceived quality of performance might be decided by professional standard pianists and what criteria might be applied, both with regards their own performances as well as the performance of others. In terms of ratings of their own performance, overall self-ratings showed a significant difference between several participants. Their evaluation behaviour in both the self-evaluation and the external evaluation were likely to be consistent in terms of how they perceived their own performances. For example, the participants who rated their own performance overall highly in the self-evaluation were likely to rank their own selected recording in the external evaluation more highly than the others did. Correspondingly, a participant who was perhaps a bit strict in the self-evaluation was likely to rank their own performance lower in the external evaluation than the others did.

In the self-evaluation phase, the criteria that performers used to evaluate performances were mainly the musical and performance elements related to tone quality, phrasing, pedalling, tempi and overall musical expression, such as storytelling and having a ‘dream character’. Even though a performance may have received higher scores on other criteria, the criteria related to musical expression were likely to be more dominant, or could be an element to raise the ranking. Also, five of the six participants gave a construct of tempo, for example slow or fast tempo. The constructs of tempo tended to be associated with interpretation. It could be thought that tempo was an important feature to describe the characteristics of the performance.

In the external evaluation, as well as self-evaluation, the most influential factor in deciding the performance quality was related to tone quality, phrasing and musical expression. Compared with the self-evaluation, none of the constructs was related to technical precision. As all participants in this study were professional pianists, it could be thought that their fundamental skill of performance did not create any concerns with technical issues. Therefore, their judgements could be focused more at a musical level, rather than on basic mechanical or technical aspects (*cf* Chaffin and Imereh, 2001). In particular, as the items for their role as external evaluators were made of their best performances, it is assumed that the quality of these particular recordings was relatively high. It would seem that technical precision was not the main focus of attention in their evaluation. It seems likely that the reason why there was no judgement evident on technical precision was the relatively high quality of performances.

Also, five of the six participants gave the construct of pedalling, for example ‘clear pedalling’ and ‘musical pedalling’. The criterion of pedalling would be piano specific as an influence on perceived performance quality and was related to phrasing or interpretation. Statistically, the criteria for self-evaluation and for in external evaluation highly overlapped for each performer (Kendall Coefficient of Concordance, w = 0.746, *p* < 0.001). It could be said that pianists in this research have similar constructs of criteria for the evaluation of piano performances, whether performed by themselves or by other pianists. In addition, several criteria given by the same performer in the external evaluation were likely to be classified closely in the cluster analysis, such as tone quality, musical expression and the quality of pedalling.

Evidence of participants’ evaluations being considered as consistent and reliable may be drawn from an agreement that the performance by Performer F was assigned high ratings and chosen as the best overall, whereas the performance by Performer D was ranked as the lowest. One of the characteristics of the performance by Performer F was its slowest tempo, which may be considered to well-reflect the characteristics of the piece “Träumerei,” which generally means “dreaming” and is sometimes translated by the term “Reverie.” Their evaluation of their own performances, namely the hidden self-evaluation in the external evaluation, could be different from the evaluation by others. This suggests that their own perspectives could be influential on their priorities and the preferences in deciding the nature of a better performance.

In this external-evaluation session, one of the six recordings was their own recording previously chosen by them as the best from the self-evaluation session. Their own performances were placed third in the set of the six recordings to be evaluated by all participants. Apart from one pianist (who declined to make a judgement), all the other performers were able to identify their own recording amongst the six used in the external evaluation session, having been informed at the end of the blind judging session that one of recordings was their own one. This finding also supports the outcomes of the research study conducted by Repp and Knoblich (2004), which demonstrated that pianists were able to recognise their own recording amongst several performances by others. According to the participants’ own reports in this current study, the reasons why they could identify their own recordings were mainly related to tone quality, phrasing, and dynamics. It would be thought that several specific features of the performance can be kept in mind by the pianist and they can feel and perceive these whilst listening Repp and Knoblich (2004) reported that the identification of self-performance was successful in their study despite of editing of tempo and dynamics. This current research suggests that sense of tone colour and phrasing could be the potentially important features in the self-identification of performance quality. Interestingly, these two elements were also demonstrated as important elements in an external evaluation session.

The performance criteria, namely viewpoints of the decision of performance quality, in both self-evaluation and external evaluation predominately overlapped in terms of musical factors. Comparing the criteria in both sessions, more than half overlapped within the same person. Performers C, D and F provided the four same criteria in both their self-evaluation and external evaluation. On the one hand, some of their written observations are slightly different in terms of wording to the provided criteria; on the other hand, some were exactly the same. Regarding Performers B and E, half of the criteria in both sessions overlapped. Performer A showed a little variety, however, with two criteria being the same in self-and external-evaluations. The result of the analysis using Kendall’s Coefficient of Concordance also showed that the categories for both criteria significantly overlapped. It can be inferred that performers’ constructed criteria for both self-evaluation and external evaluation are relatively associated.

The results from the current research empirically demonstrated that criteria related to tone quality and musical expression appear to be the most dominative components in deciding the overall quality of performance in both the self-evaluation and the external evaluation phases. Focused on the external evaluation, the most assigned element was phrasing (7/36 items) and then tone quality (6/36 items). Particularly when in the role of external evaluator, tone quality and overall flow were the most associated in the decision to award higher rankings. These results are supported, at least in part, by extant literature, such as the studies by Russell (2010) and Thompson et al. (1998). Russell (2010) found that the component of musical expression had a significantly direct effect on the overall perception of quality. Thompson et al. (1998) found that an overall assessment was strongly related to the evaluation of musical expression, phrasing and right-hand expression. Tempo could be important to identify the quality of performance in this study; however, it was not the main element to emerge in determining the quality of the performances. This finding agreed with the outcome of the study by Thompson et al. (1998) which reported that tempo could be important for identifying performance quality; however, it was not highly associated with overall preferences.

At the opening recording session, all performers reported a perception that the ‘best’ performance was in the latter half of their playing set, especially the fifth and sixth versions. In the self-evaluation session with playback, the participants were still likely to choose the best performance from the latter half of the session. These results support other research suggesting that the order of performance can be influential in evaluation (Duerksen, 1972; Flôres and Ginsburgh, 1996). However, it may be said that even these professional pianists did not always make a decision of their best performance concordantly in both recording time (just after the performances) and later at the time of self-evaluation.

This research study suggests that the participant professional pianists did not always consistently evaluate their own performance as others did. However, in terms of the relationship between the roles of self-evaluation and external evaluation by the same performer, the tendencies evidenced within self-evaluation could be found in the context of the role of external evaluator. These interactions indicated that a self-constructed tendency of evaluation is the basis of specific and individual attitudes when deciding the comparative quality of musical performances, by self and others.

## Data availability statement

The original contributions presented in the study are included in the article/Supplementary material, further inquiries can be directed to the corresponding author.

## Ethics statement

The studies involving human participants were reviewed and approved by The Institute of Education, formerly University of London, now University College London. The patients/participants provided their written informed consent to participate in this study.

## Author contributions

The research cited originally formed part of the doctoral studies of YM. Supervised and supported by GW. The methodology was designed collaboratively and the fieldwork was undertaken by YM. All authors contributed to the article and approved the submitted version.

## Funding

This research study was partly supported by The Arnold Bentley New Initiatives Fund by SEMPRE.

## Conflict of interest

The authors declare that the research was conducted in the absence of any commercial or financial relationships that could be construed as a potential conflict of interest.

## Publisher’s note

All claims expressed in this article are solely those of the authors and do not necessarily represent those of their affiliated organizations, or those of the publisher, the editors and the reviewers. Any product that may be evaluated in this article, or claim that may be made by its manufacturer, is not guaranteed or endorsed by the publisher.

## Footnotes

^1^Ward’s method, namely ‘Ward’s minimum variance method’, was originally proposed by Joe H. Ward Jr. in 1963. In hierarchical cluster analysis, this method is one of most commonly used approaches that combines objects "whose merger increases the overall within-cluster variance to the smallest possible degree, are combined" (Mooi and Sarstedt, 2011, p. 252).
