# Supplementary material for: Decisions on the quality of piano performance: Evaluation of self and others
Source: Front Psychol. 2022 Nov 17;13:954261. doi: 10.3389/fpsyg.2022.954261 (PMC9714486; doi:10.3389/fpsyg.2022.954261)
Supplement: Supplementary file 1 [file Data_Sheet_1.docx]

Supplementary Material

Supplementary Table 1. Overall rating on external evaluation by each participant

| Adjudicator | A | B | C | D | E | F |
| --- | --- | --- | --- | --- | --- | --- |
| Mean | 5.33 | 6.33 | 5.67 | 6.11 | 5.69 | 6.03 |
| SD | 1.72 | 3.10 | 2.50 | 2.22 | 2.85 | 2.14 |

Supplementary Table 2. The results of ranking at the external evaluation with converted points

|  | Evaluators | | | | | | Total  points | SD | Mean | Total  Ranking |
| --- | --- | --- | --- | --- | --- | --- | --- | --- | --- | --- |
|  | **A** | **B** | **C** | **D** | **E** | **F** |  |  |  |  |
| Performance by A | **2** | 2 | 4 | 4 | 2 | 4 | 18 | 1.10 | 3.0 | 5 |
| Performance by B | 3 | **6** | 2 | 1 | 4 | 3 | 19 | 1.72 | 3.2 | 4 |
| Performance by C | 4 | 5 | **3** | 5 | 3 | 5 | 25 | 0.98 | 4.2 | 2 |
| Performance by D | 1 | 1 | 1 | **3** | 1 | 1 | 8 | 0.82 | 1.3 | 6 |
| Performance by E | 5 | 3 | 5 | 2 | **5** | 2 | 22 | 1.51 | 3.7 | 3 |
| Performance by F | 6 | 4 | 6 | 6 | 6 | **6** | 34 | 0.82 | 5.7 | 1 |

The numbers with bold and underlines = the evaluator’s own performance.
